# Impact of pharmaceutical intervention on the use of intravenous antibiotics in patients with bacterial upper respiratory tract infections: protocol for a cluster-randomized controlled trial

**DOI:** 10.3389/fpubh.2026.1742217

**Published:** 2026-03-10

**Authors:** Jiali Zhang, Song-gao Lou, Jianbo Chu, Lingdi Zhang, Qihan Wu, Yanyan Xu, Yu Dong, Taotao Hu, Meng Huo, Ping Yan, Rui Shao, Jiefang Zhou, Zhouhong Zhan, Mengdi Zhang, Chaofeng Ji, Lisha Lin, Jinming Wang, Shuangshuang Zhou, Min He, Liping Deng, Jianghuan Ke, Jianan Wang, Junhong He, Jie Chen, Shengjiang Wang, Zhuanghua Rui, Tongtong Yang, Yuye Huang, Yue Zhang, Lu Ke, Tao Chen, Huifang Jiang, Meng Chen, Yangmin Hu, Haibin Dai

**Affiliations:** 1 Department of Pharmacy, Second Affiliated Hospital, Zhejiang University School of Medicine, Hangzhou, China; 2 Research Center for Clinical Pharmacy, Zhejiang University, Hangzhou, China; 3 Department of Pharmacy, Sheng Zhou Hospital of Traditional Chinese Medicine, Shaoxing, China; 4 Department of Pharmacy, The Affiliated Yangming Hospital of Ningbo University, Ningbo, China; 5 Department of Pharmacy, Affiliated Xiaoshan Hospital, Hangzhou Normal University, Hangzhou, China; 6 Department of Pharmacy, The First People’s Hospital of Lin’an District, Hangzhou, China; 7 Department of Pharmacy, The Fifth Affiliated Hospital, Wenzhou Medical University, Lishui, Zhejiang, China; 8 Department of Pharmacy, People’s Hospital of Kaihua, Quzhou, China; 9 Department of Pharmacy, Shaoxing Second Hospital, Shaoxing, China; 10 Department of Pharmacy, The First Affiliated Hospital of Wenzhou Medical University, Wenzhou, China; 11 Department of Pharmacy, Yuyao Maternal and Child Health Hospital, Ningbo, China; 12 Quzhou Hospital of Traditional Chinese Medicine, Quzhou, China; 13 Department of Pharmacy, Shaoxing Hospital of Traditional Chinese Medicine, Shaoxing, China; 14 Affiliated Hospital of Shaoxing University, Shaoxing, China; 15 Department of Pharmacy, Shaoxing Central Hospital, Shaoxing, China; 16 Department of Pharmacy, De Qing People's Hospital, Huzhou, China; 17 Department of Pharmacy, The First People's Hospital of Yuhang District, Hangzhou, China; 18 Department of Pharmacy, Taizhou Municipal Hospital (Taizhou University Affiliated Municipal Hospital), School of Medicine, Taizhou University, Taizhou, China; 19 Department of Pharmacy, Tiantai People's Hospital, Taizhou, China; 20 Department of Pharmacy, The First People's Hospital of Xiaoshan District, Hangzhou, China; 21 The First People's Hospital of Pinghu, Jiaxing, China; 22 Department of Pharmacy, Jinhua Municipal Central Hospital, Jinhua, China; 23 Department of Pharmacy, Ningbo No.2 Hospital, Ningbo, China; 24 Department of Pharmacy, Ningbo No.6 Hospital, Ningbo, China; 25 Department of Pharmacy, Wenzhou People's Hospital, Wenzhou, China; 26 The Second People's Hospital of Tongxiang, Tongxiang, China; 27 The Second People's Hospital of Jiashan, Jiaxing, China; 28 Department of Pharmacy, The First Affiliated Hospital of Ningbo University, Ningbo, China; 29 The People's Hospital of Cangnan, Wenzhou, China; 30 The People's Hospital of Changxing, Huzhou, China; 31 Department of Critical Care Medicine, Jinling Hospital, Medical School of Nanjing University, Nanjing, China; 32 Global Health Trials Unit, Liverpool School of Tropical Medicine, Liverpool, United Kingdom

**Keywords:** bacterial upper respiratory tract infections, emergency department, intravenous antibiotic administration, pharmaceutical intervention, pharmacist

## Abstract

**Background:**

Overuse of antibiotics among patients with upper respiratory tract infections (URTIs) is a worldwide problem. In China, approximately 70% of outpatients with URTIs are treated with antibiotics, often via intravenous infusion. This study aimed to evaluate whether a pharmacist-led multidimensional intervention reduces the use of intravenous (IV) antibacterial drug infusion among patients with bacterial URTIs.

**Methods and analysis:**

This study employed a pragmatic, parallel controlled, cluster-randomized superiority trial design. Outcome assessment and data analysis were conducted in a blinded manner, while treatment administration remained unblinded. A total of 28 hospitals in Zhejiang Province, China, were randomly allocated in a 1:1 ratio. In the intervention arm, a multidimensional intervention embedded in routine emergency treatment will be applied, involving both doctors and patients. The interventions included systematic physician training, clinical decision support cards, and printed educational materials for patients. Patients admitted to the sites assigned to the control arm will receive usual care at the discretion of treating physicians. The primary outcome was the rate of intravenous antibacterial drug infusion during the index admission. Secondary outcomes included the duration of URTI symptoms, adverse events, proportion of eligible patients who received subsequent antibiotics, frequency of re-consultation, and hospitalization within the follow-up period. The final follow-up was completed by 14 days post-discharge. Participants will be included from August 1, 2025, to January 31, 2026.

**Conclusion:**

This study will demonstrate the feasibility and potential impact of a pharmacist-led, multidimensional intervention aimed at reducing IV antimicrobial use in patients with bacterial URTIs.

**Clinical trial registration:**

ClinicalTrials.gov, identifier NCT06620341.

## 1 Introduction

Antibiotic misuse remains a global challenge (1), especially in China (2, 3). These practices increase healthcare costs and accelerate the development of antimicrobial resistance (AMR) (4). China has one of the fastest-growing AMR problems in the world (5). An epidemiological survey encompassing 156 public hospitals across 30 Chinese provinces demonstrated that 93.1% of hospitalized patients received intravenous (IV) infusions in 2016 (6). Among these, the use of broad-spectrum antimicrobials is particularly prevalent. Overuse of IV antibiotics is a critical challenge in global antimicrobial stewardship efforts (7, 8), particularly in emergency department (ED) settings, where empirical treatment decisions are frequently made under time pressure (9). Acute upper respiratory tract infections (URTIs), predominantly of viral (70–80%) or bacterial (20–30%) origin, are the most prevalent infections worldwide (10). Despite the predominant viral etiology of URTIs, studies have reported that 50–70% of affected patients receive antimicrobial prescriptions (11, 12). A 2017 study reported that in rural Chinese healthcare settings, IV broad-spectrum antimicrobial usage rates for pediatric URTIs reached 65%, 43%, and 33% at county, township, and village levels, respectively (13). It was found in another trial across 20 Chinese primary care institutions that more than 77% of URTI prescriptions required intravenous drug injection or infusion (14). Overuse of IV infusions exposes patients to unnecessary drug-related side effects and increases the financial burden. According to the 2024 Annual Report of China’s National Adverse Drug Reaction Monitoring System, IV administration accounted for 57.2% of the 2,597,000 reported adverse drug reactions, substantially exceeding the proportion attributed to oral administration (33.1%) (15).

Current evidence suggests that about 41% of antibiotic prescriptions for URTIs may be unnecessary (16), with IV formulations being disproportionately overused compared to oral alternatives (13). IV therapy should be considered inappropriate in any patient who can eat and drink, and in those who are treated with IV medication for which an oral alternative exists. Several national policies have been issued by the Ministry of Health in China, including a policy that has piloted a ban on IV antibiotic use for outpatients in secondary and tertiary hospitals since 2016 (17). A study of secondary and tertiary hospitals in China revealed that this policy has diverted patient flow from the outpatient department to the ED, inpatient wards, and primary care for IV antibiotic prescriptions (18). Despite established guidelines promoting oral therapy, implementation gaps persist due to various factors, including diagnostic uncertainty, patient expectations, and habitual prescription patterns (19, 20).

Pharmacist-led interventions have demonstrated promise in optimizing antibiotic use across multiple healthcare settings (21, 22). A 2024 systematic review revealed that pharmacist-led antimicrobial stewardship programs improved antimicrobial prescriptions while reducing surgical site infections (SSIs) (23). However, few studies have examined the impact of pharmaceutical interventions on the IV-to-oral conversion of URTIs in EDs, where workflow pressures and acuity perceptions create unique implementation challenges.

This cluster-randomized controlled trial protocol describes a novel approach to address this evidence gap. We hypothesized that a multifaceted pharmaceutical intervention incorporating (1) clinician education, (2) clinical decision support cards, and (3) patient education would significantly reduce unnecessary IV antibiotic administration for bacterial URTIs in the ED. The study design aligns with China’s National Action Plan to Contain Antimicrobial Resistance (2022–2025), while providing a transferable model for antimicrobial stewardship in resource-varied healthcare systems.

These findings may inform national policies and international best practices for antimicrobial stewardship during emergency care by establishing the efficacy of pharmacist-driven interventions in this high-use clinical scenario. This pragmatic trial design ensures that the results may be directly applicable to real-world ED operations, potentially offering a scalable solution to mitigate AMR risks while maintaining clinical outcomes.

## 2 Methods and analysis

### 2.1 Objectives

The primary objective of this study is to assess the effectiveness of a multifaceted pharmaceutical intervention on the rate of intravenous antibiotic administration in patients with bacterial upper respiratory tract infections in the emergency department, compared to a standard treatment without pharmaceutical intervention. Secondary objectives include evaluating the pharmaceutical intervention’s effects on the duration of URTI symptoms, the incidence of adverse events, and the rates of subsequent antibiotic use, healthcare re-engagement (outpatient or emergency department), or being hospitalized within 14 days of follow-up.

### 2.2 Trial design

**Figure 1:**
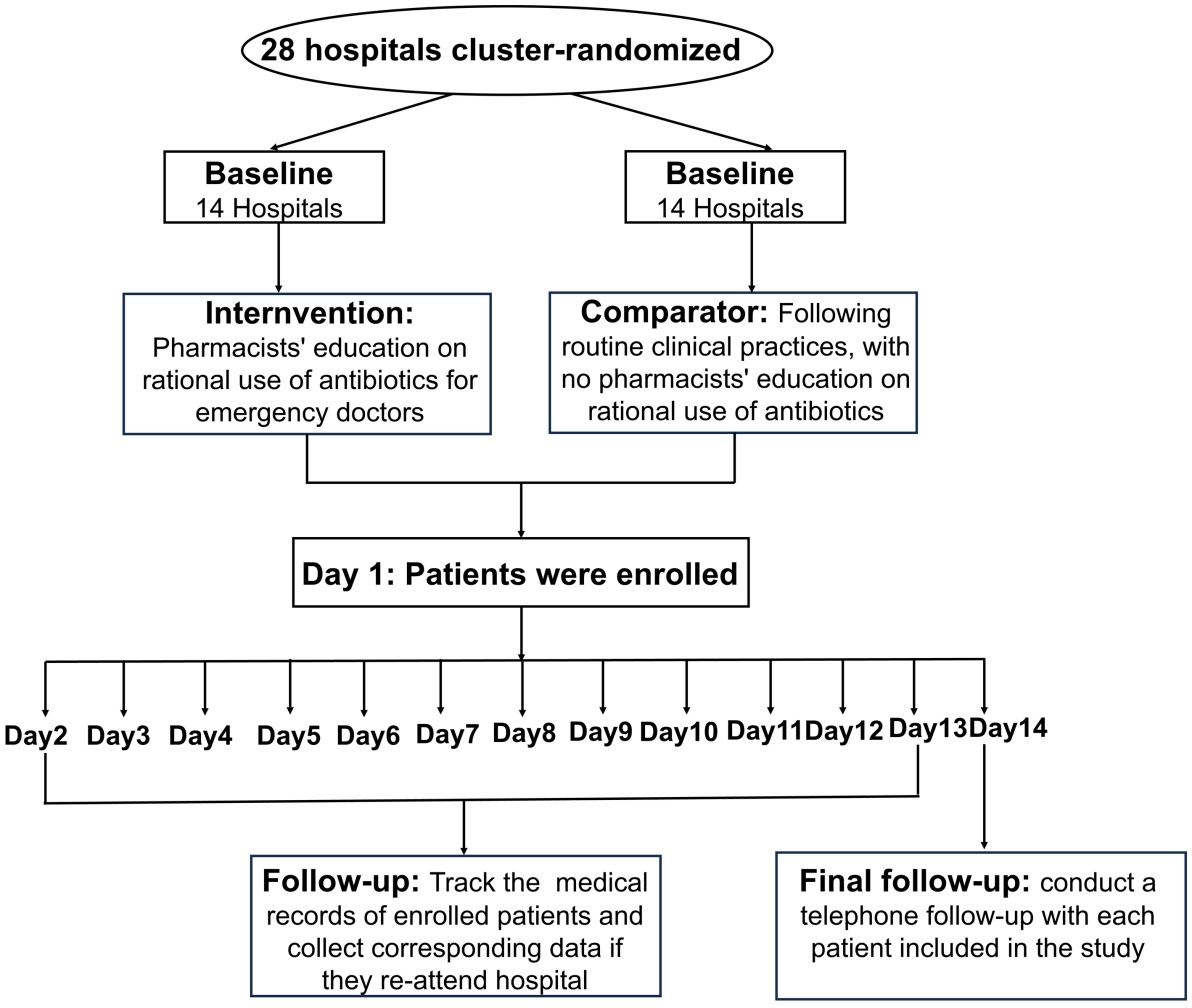
Study flow diagram.

This is a pragmatic, parallel-group, cluster-randomized controlled trial, stratified by hospital tier classification, comparing pharmaceutical interventions with usual care in 28 participating hospitals. This study was designed according to the Standard Protocol Items: Recommendations for Interventional Trials (SPIRIT) guidelines (2025). The flowchart of the study is presented in Figure 1. A cluster-randomized design was chosen because some aspects of the intervention made it infeasible to allocate doctors within the same hospital to different groups due to logistical reasons and potential contamination risks.

### 2.3 Study setting

This study was sponsored by the Second Affiliated Hospital of Zhejiang University School of Medicine, a tertiary care hospital in eastern China. All 28 participating hospitals, selected based on eligibility criteria, were located in Zhejiang Province, an economically developed region in China. Although participating hospitals had inpatients, we only considered outpatients in the ED because inpatients often present with multiple comorbidities, making it difficult to assess rational prescriptions for URTIs.

### 2.4 Eligibility criteria

#### 2.4.1 Eligibility criteria for clusters

Hospitals were eligible to participate in the trial if they treated patients with URTI in the ED and had not previously implemented specific interventions to reduce the rate of IV antibiotic administration. Fifteen hospitals were excluded from the study due to significantly lower baseline rates of IV antibiotic use for URTIs than peer institutions, with rates consistently remaining at < 50%.

#### 2.4.2 Eligibility criteria for participants

Eligible patients had to satisfy all of the following criteria: aged ≥ 18 years, had a URTI diagnosis during the baseline and intervention periods, with laboratory tests indicating white blood cells > 10 × 10^9^/L or C-reactive protein (CRP) ≥ 10.0 mg/L, and required the use of antimicrobial agents with at least one focal sign or symptom. Qualifying manifestations included (1) fever, (2) cough, (3) rhinitis (sneezing, nasal congestion, or rhinorrhea), (4) pharyngitis (sore throat), (5) dyspnea, (6) wheezing, or (7) chest pain.

Exclusions would apply to patients with other infections in addition to URTIs; patients who require hospitalization or referral to a higher-level medical institution; individuals unable to tolerate oral medications; special populations, including those with neutropenia, bone marrow suppression, active radio-chemotherapy, immunosuppression, HIV/AIDS, primary immunodeficiency, pregnancy, or severe psychiatric disorders and patients who were not prescribed antimicrobial agents during this visit.

### 2.5 Sample size

Based on our previous survey, the current antimicrobial infusion rate among patients with URTIs is estimated to be 50%–70%. A sample size of 1,400 participants per group was calculated to achieve 82% power for detecting a difference of 0.12 in group proportions. This was based on sampling 14 clusters of 100 subjects each in control group and 14 clusters of 100 subjects each in the intervention group. The proportion in intervention group is assumed to be 0.48 under the null hypothesis and 0.6 under the alternative hypothesis. The test statistic used is the two-sided Z-Test (unpooled), with an intracluster correlation of 0.04 and a significance level of 0.05. To account for a potential 10% dropout rate, the sample size was increased to 1,556 participants per group.

### 2.6 Randomization

A total of 28 hospitals were eligible for the trial, comprising 9 Tier-3 Grade-A hospitals, 15 Tier-3 Grade-B hospitals, and 4 Tier-2 Grade-A hospitals, based on China’s tiered hospital classification system. Under this system, hospitals are categorized into three tiers (Tier-1 to Tier-3), with Grade-A representing the highest service quality within each tier. Randomization was performed at the hospital level. All participating institutions were stratified by hospital grades to ensure balanced cluster distribution between the study arms. Hospitals within each stratum were randomly allocated to either the intervention or control group in a 1:1 ratio, using a computer-generated sequence. To maintain allocation concealment, randomization was performed after obtaining each hospital’s consent to participate.

To minimize observer bias, randomization was conducted by a member of research team who was not involved in recruitment or participant enrolment. Due to the interventional nature, masking of researchers and participants was impossible; however, telephone follow-up personnel and trial statisticians remained blind to cluster allocation until the trial was concluded. Regular meetings were conducted among the investigators to monitor research progress and effectively address any issues in this study.

### 2.7 Interventions

This multidimensional intervention strategy where pharmacists actively engage across three synergistic domains to optimize antibiotic use. It includes providing structured education and performance feedback to clinicians, implementing evidence-based clinical decision support tools to standardize prescribing, and developing and deploying patient-centered educational materials to facilitate informed decision-making. This intervention aligns with national antibiotic stewardship policies and local health authority regulations, specifically developed to improve appropriate IV antibiotic prescriptions in emergency settings.

#### 2.7.1 Training program

Pharmacists conducted monthly educational sessions for ED healthcare providers on the rational use of antimicrobial infusions at the beginning of each month, with actual IV infusion rate feedback provided to the clinical staff every two weeks. The templates of the main training materials adopted by each intervention center are provided in the Supplementary Appendix SA1. The training sessions had been minuted. Final prescription decisions, including antibiotic selection, duration, and route of administration, were determined based on the clinician’s clinical judgment. Physicians were mandated to transfer patients to tertiary care facilities if deemed necessary.

#### 2.7.2 Clinical decision support

Evidence-based clinical decision support cards were implemented to optimize antibiotic prescription practices among the clinicians. Physicians conducted comprehensive clinical assessments of patients with CRP levels of > 10 mg/L before initiating antibiotic therapy. Oral antibiotics with high bioavailability are preferred for mild-to-moderate infections, and parenteral administration is limited to cases that meet the following predefined criteria: (1) intolerance to oral medications, including dysphagia and persistent vomiting; (2) clinically significant malabsorption disorders, including severe diarrhea, gastrointestinal disorders, or malabsorption syndromes; (3) lack of suitable oral alternatives; (4) life-threatening infections requiring urgent treatment, such as sepsis and necrotizing pneumonia; (5) poor adherence to oral treatment regimens. Emergency medical staff across all intervention centers utilized a standardized pocket knowledge card, details of which are available in the Supplementary Appendix SA2.

#### 2.7.3 Patient education

Structured patient education materials were systematically developed and distributed as part of a comprehensive antimicrobial stewardship initiative to promote awareness of appropriate IV antibiotic use. The educational material, titled “Standardized Intravenous Antibiotic Therapy,” employed health literacy principles to communicate three key messages: (1) indications for appropriate IV antibiotic use; (2) evidence-based recommendations for antimicrobial agents with equivalent efficacy in oral and IV formulations; (3) potential risks associated with unnecessary IV administration. The visually optimized educational materials were distributed by attending physicians during clinical consultations to ensure direct patient engagement at the point of care. The presentation format of the patient education manual is provided in the supplementary materials of this paper (Supplementary Appendix SA3).

Content development followed a rigorous process, including review by infectious disease specialists, clinical pharmacists, and health communication experts, to ensure accuracy and accessibility.

### 2.8 Follow-up

This prospective study implemented a two-phase follow-up protocol to optimize data completeness. Trained research staff at each site performed standardized medical record reviews 7- and 14-day post-discharge. All subsequent visits for bacterial URTIs were systematically recorded in an electronic data capture system using standardized case report forms. At the 14-day follow-up, telephone interviews to assess clinical outcomes were conducted by centrally assigned, trained interviewers blinded to participants’ group allocation. Prior to the follow-up period, we developed an online structured questionnaire for telephone surveys, with logic jumps and mandatory fields to minimize manual data entry errors. All interviewers completed a standardized training session before follow-up initiation to ensure consistency in survey administration. This study exclusively used routine clinical data without requiring additional clinical or laboratory tests, minimizing the participant burden and preserving healthcare resources. Patients will be included from August 1, 2025, to January 31, 2026.

### 2.9 Outcomes

#### 2.9.1 Primary outcome

The primary outcome was the initial antibacterial drug infusion rate in the two study arms, defined as the proportion of eligible patients who received IV antibiotic therapy, assessed from randomization until the end of the intervention (Table 1).

**Table 1:** Primary and secondary outcomes.

Hierarchy	Trial outcome	Definition	How it was measured
Primary	Infusion rate of antibacterial drugs	Infusion of antibacterial drugs	Hospital records
Secondary	Disease maintenance	Days of respiratory tract infection symptoms keep	Questionnaire by telephone follow-up
Secondary	Adverse drug reaction	Allergic reaction, rash, or pruritus	Questionnaire by telephone follow-up
Secondary	Disease recurrence within 14 days	Patients re-attend the outpatient clinic or the ED of the hospital for treatment	Questionnaire by telephone follow-up
Secondary	Disease recurrence within 14 days	Patients who did not re-attend the outpatient clinic or emergency but re-used the antibiotic infusion after the first visit in the emergency	Questionnaire by telephone follow-up
Secondary	14-day unplanned hospitalization	Patients be hospitalized	Questionnaire by telephone follow-up

#### 2.9.2 Secondary outcomes

We estimated the effects of this intervention on several secondary outcomes.

1. The duration of URTI symptoms was verified by all random patient exit interviews via phone call follow-up after 14 days.
2. Adverse events, severe adverse events, and adverse events related to antibiotics include allergic reactions, rashes, and pruritus.
3. The proportion of eligible patients who received subsequent antibiotics for any reason within 14 days of follow-up was verified by all randomized patient exit interviews via telephone follow-up. Subsequent antibiotic use was defined as any antibiotic use within 14 days of follow-up, excluding initial antibiotic prescriptions.
4. The proportion of eligible patients with URTIs who re-attended outpatient clinics or emergencies within 14 days, and whether antibiotics were prescribed, compared to intervention hospitals and controls.
5. The proportion of eligible patients who were hospitalized within 14 days was verified by all randomized patient exit interviews via phone call follow-up after 14 days, excluding patients referring to the initial ED visit.

### 2.10 Data collection and management

For every patient, quantitative and qualitative data were obtained from electronic medical records, where available, and clinical outcomes were derived from telephone follow-up evaluations. A secure web-based database was established for data collection, with access restricted to principal investigators and site co-investigators. Collected variables included consultation date, demographic characteristics (gender and age), diagnosis, prescribed medications, body temperature, and relevant laboratory results. Each participant was assigned a unique study number to ensure anonymity throughout the data entry and analysis.

All site investigators entered and modified the data using the original source-verified records. The coordinating center conducted regular data audits to verify the accuracy and protocol compliance. The identified discrepancies triggered a standardized query process for clarification and correction by the designated staff. Before study initiation, the principal investigator conducted standardized training for all co-investigators to ensure consistent data collection procedures. The database incorporates range checks and value validation to minimize data entry errors.

### 2.11 Statistical analysis

This study will adhere to the CONSORT guidelines for cluster-randomized trials. Based on the intention-to-treat principle, a full analysis set (FAS) will be applied to all randomized subjects at all participating hospitals. Per-protocol analysis of the primary endpoint included only participants who underwent interventions. FAS will be used to study baseline characteristics and primary outcome comparison. A two-sided significance level of 5% will be used to determine the statistically significant results.

Pre-specified subgroup analyses for the primary endpoint will be performed as part of the primary analysis. For between-group comparisons, generalized linear models will be used, with treatment group, hospital level as fixed effects and healthcare center as a random effect. Relative risks (RRs)and risk differences with confidence intervals (CIs) will be calculated. Symptom duration will be assessed using Kaplan–Meier curves with 95% CIs. Formal comparisons between the two groups will beperformed using the Cox proportional hazards model, with the treatment group and hospital level as fixed effects and healthcare center as a Gaussian random effect (frailty). A detailed statistical analysis plan will be prepared before data lock.

#### 2.11.1 Handling of missing data

For the missing primary outcome measure, we will assume that the data are missing at random (MAR) and thus “ignore” in the primary analysis. However, if > 5% of the primary outcome measures that should have been available for analysis are missing, a sensitivity analysis will be conducted in addition to a primary MAR analysis.

For missing baseline prognostic variables, the mean value calculated from the observed non-missing instances of the baseline prognostic variable will be used for the imputation. These imputed means will be calculated based on pooled data from both study groups and will notbe computed within groups using group-specific data. If imputation is required as a baseline prognostic variable, the proportion of cases with initially missing values will be reported.

### 2.12 Ethics and dissemination

This study was reviewed and approved by the medical ethics committee of the Second Affiliated Hospital, Zhejiang University School of Medicine, China (Approval No. 2024–0799), as well as by the ethics committees of all participating centers. All study sites were approved by the local institutional ethics committee. The requirement for written informed consent from the patients was waived by the ethics committee to ensure minimal disruption to routine practice. The results of this trial will be published in peer-reviewed journals and presented at conferences.

An independent Trial Steering Committee (TSC) oversees this study. TSC, composed of clinical experts, statisticians, and independent advisors, monitors the trial progress, evaluates protocol adherence, addresses significant deviations, and makes final decisions regarding trial modifications. The committee ensured scientific rigor and ethical compliance through an independent review.

## 3 Discussion

This cluster-randomized trial demonstrated the feasibility and potential impact of a pharmacist-led, multidimensional intervention aimed at reducing IV antimicrobial use in ED patients with bacterial URTIs. By integrating clinician education, evidence-based decision support, and patient education, the intervention aligns with global antimicrobial stewardship (AMS) priorities, while addressing context-specific challenges in China’s healthcare system.

To the best of our knowledge, this is the first study to investigate the impact of pharmacist-led interventions on decreasing irrational IV antimicrobial therapy in China. Pharmacist-led AMS programs have been effective in enhancing the appropriateness of antibiotic selection, timely administration, and reducing the duration of antimicrobial therapy. Elnour AA et al. reported that the clinical pharmacist’s implementation of the surgical antimicrobial prophylaxis protocol revealed a significant reduction in SSIs from 42.5% to 25.7% in the intervention group compared to the reduction from 57.5% to 44.2% the control group (*p* = 0.001) (24). Zhou et al. (25) revealed that the reasonable antibiotic rate increased significantly in terms of usage and dosage (96.6% versus 83.9%, *p* < 0.001), timing of administration (94.5% versus 78.4%, *p* < 0.001), and medication duration (64.4% versus 37.7%, *p* < 0.001) in patients undergoing orthopedic internal fixation during the pharmacist intervention period. This intervention framework, which targets prescriber behavior and patient perceptions, reflects the growing recognition that AMS success requires addressing the systemic and cultural drivers of antimicrobial misuse. The emphasis on IV-to-oral conversion criteria resonates with international guidelines advocating step-down therapies for stable infections. However, this approach uniquely adapts these principles to China’s high IV use setting. However, the 65% baseline IV antibiotic rate for pediatric URTIs reported in rural China underscores the entrenched prescribing norms that our intervention sought to disrupt (13). This study further demonstrated pharmacists’ unique capacity to translate national AMS policies into context-specific protocols. This adaptation of the IV-to-oral conversion criteria to accommodate China’s high patient volumes and diagnostic uncertainty exemplifies how pharmacists’ expertise can balance safety with feasibility. Nevertheless, sustainable impact requires institutionalizing pharmacists’ authority in antimicrobial decision-making, a cultural shift still underway in many Chinese hospitals.

The trial’s pragmatic design enhances real-world applicability, as evidenced by its integration into routine workflows, without requiring additional diagnostics. This scalability aligns with China’s 2025 National Medical Quality Goals, which prioritize feasible interventions to curb IV overuse. If the anticipated 12% relative reduction in IV antibiotic initiation is achieved, it could translate to substantial public health benefits, given China’s annual URTI burden. Extrapolating from this sample size assumption (n = 3,112), a 12% absolute reduction would eliminate approximately 1,200 unnecessary IV courses per 10,000 patients, mitigating associated adverse events, such as infusion reactions, AMR selection, and healthcare costs. Furthermore, the inclusion of patient education materials counteracts the pervasive misconceptions linking IV therapy to enhanced efficacy. This approach complements recent policy shifts toward value-based care by empowering patients to question unnecessary infusions.

This study has several limitations. First, it was not a double-blind study. Although efforts have been made to control bias, the telephone follow-up personnel and trial statisticians’ blinded design of this study could not avoid bias. Second, because this was a cluster-randomized trial, variations in baseline infusion rates across different hospitals may result in heterogeneity in outcomes. To minimize the impact of baseline infusion rate variation, we stratified randomization by hospital grade and baseline proportion of patients with URITs prescribed IV antibiotics. However, the exclusion of hospitals with baseline IV rates of < 50% limits the generalizability to low-performing settings. Third, the study period coincided with the nationwide implementation of AMS policies and increased awareness of IV antibiotic overuse, which may have introduced confounding factors. As such, it is difficult to disentangle the specific effect of our program from these concurrent secular trends. Finally, the 14-day follow-up window may underestimate late complications or AMR development, and long-term studies are needed to assess durability.

## 4 Conclusion

The protocol was carefully designed to ensure a detailed and feasible pharmacist-led multidimensional intervention aimed at reducing IV antimicrobial use. By targeting both prescribers and patients, the intervention addresses the systemic and cultural drivers of IV antimicrobial overuse while maintaining therapeutic efficacy. If successful, this model not only validate the critical role of pharmacists in promoting responsible antimicrobial use but also establish a transferable model for antimicrobial stewardship. Therefore, this protocol will provide a template for adapting AMS strategies for use in settings with high IV use.
