# Supplementary material for: Impact of pharmaceutical intervention on the use of intravenous antibiotics in patients with bacterial upper respiratory tract infections: protocol for a cluster-randomized controlled trial
Source: Front Public Health. 2026 Mar 10;14:1742217. doi: 10.3389/fpubh.2026.1742217 (PMC13008920; doi:10.3389/fpubh.2026.1742217)
Supplement: Supplementary file 1 [file Data_Sheet_1.PDF]

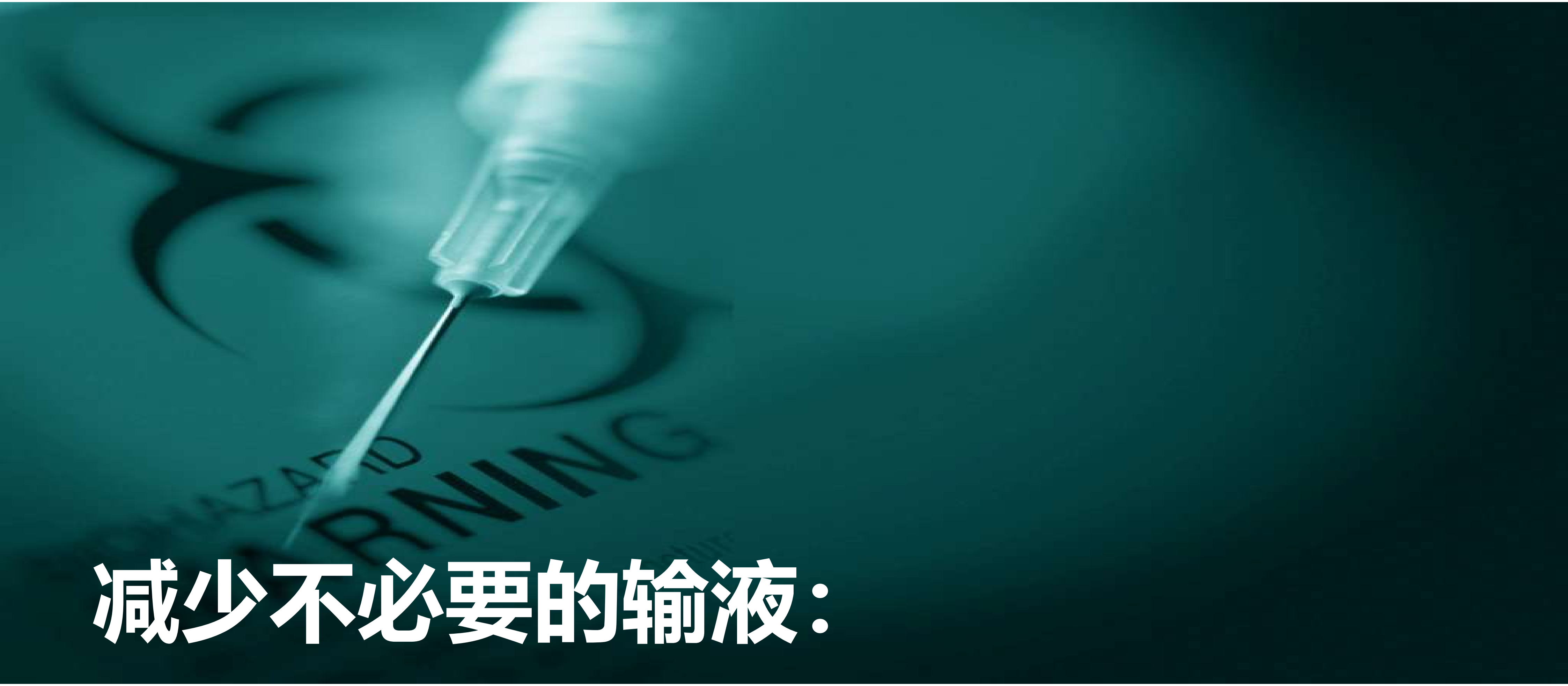

减少不必要的输液：

急诊抗菌药物合理使用策略

背景与现状

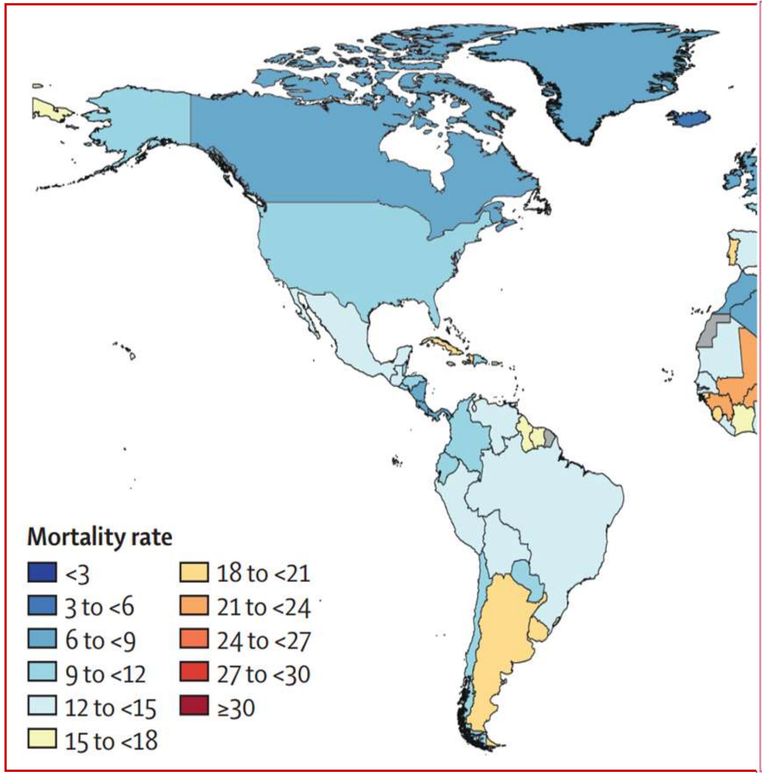

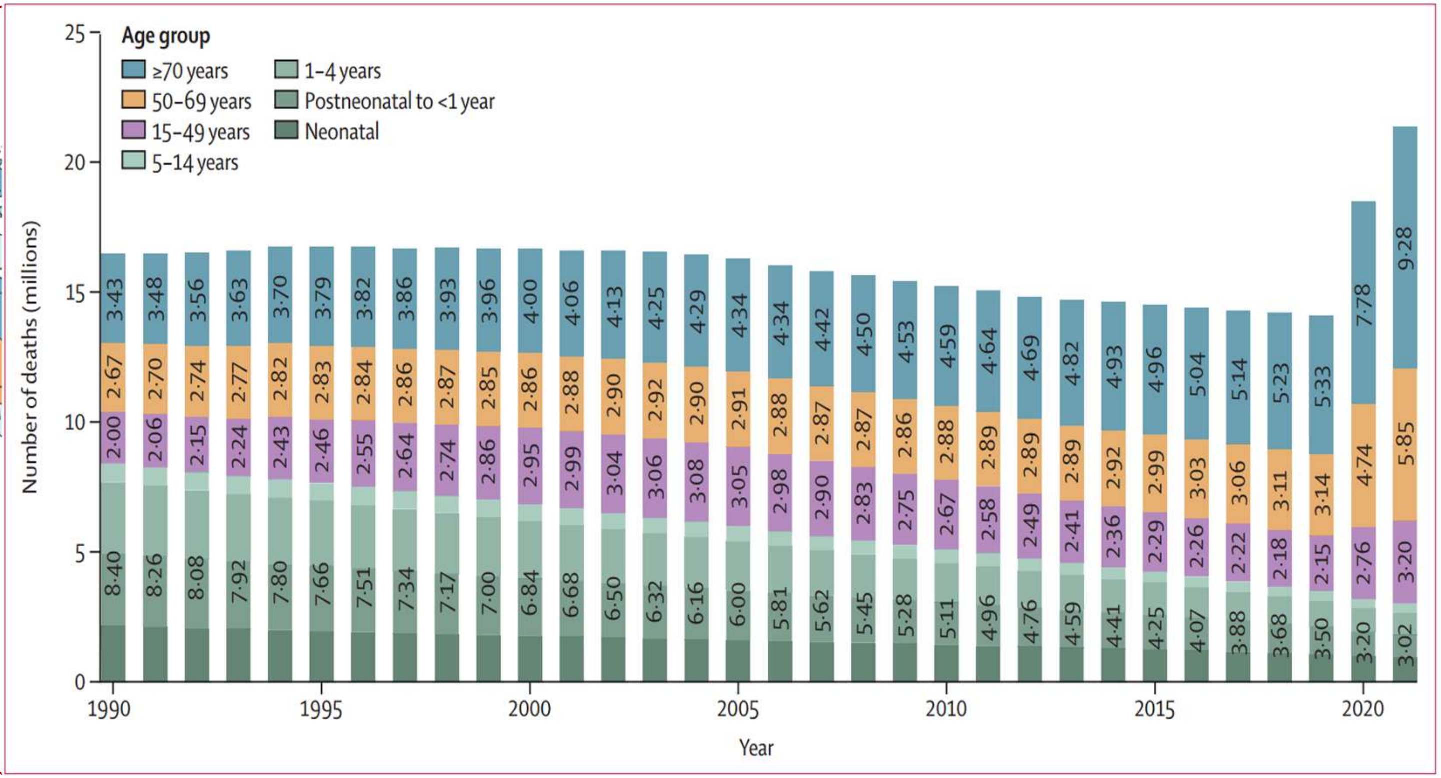

抗菌药物的过度使用促进了微生物耐药性的发展

GBD 2021 Antimicrobial Resistance Collaborators. Lancet, 2024, 404(10459): 1199-1226.

背景与现状

- 根据WHO的统计数据，全球每年输液约160亿次，**中国占了约50亿次（＞30%），是世界最大的“输液大国”**
- 国家发改委曾在全国会议上表示，我国医疗输液一年约104亿瓶，平均到13亿人口，相当于每个**中国人一年挂8个吊瓶，**远远高于国际上2.5-3.3瓶的平均水平
- 在我国基层门急诊患者的输液率平均为30%，最高达60%-70%
- 住院病人输液率更高，我国一些医院高达90%，而国外约45%
- 据WHO的一项数据显示，中国70%的输液是不必要的

Injection safety-World Health Organization;
http:/ /www.china.com.cn/news/2010-12 /24 / content _21609895.htm

背景与现状

静脉输液 vs 口服给药

01 **静脉输液风险高于口服**：过敏反应、静脉炎、感染等

02 静脉用药的便利性较口服给药差

03 从经济学角度同类静脉给药成本一般高于口服给药

WHO指导原则

能口服不肌注，

能肌注不输液

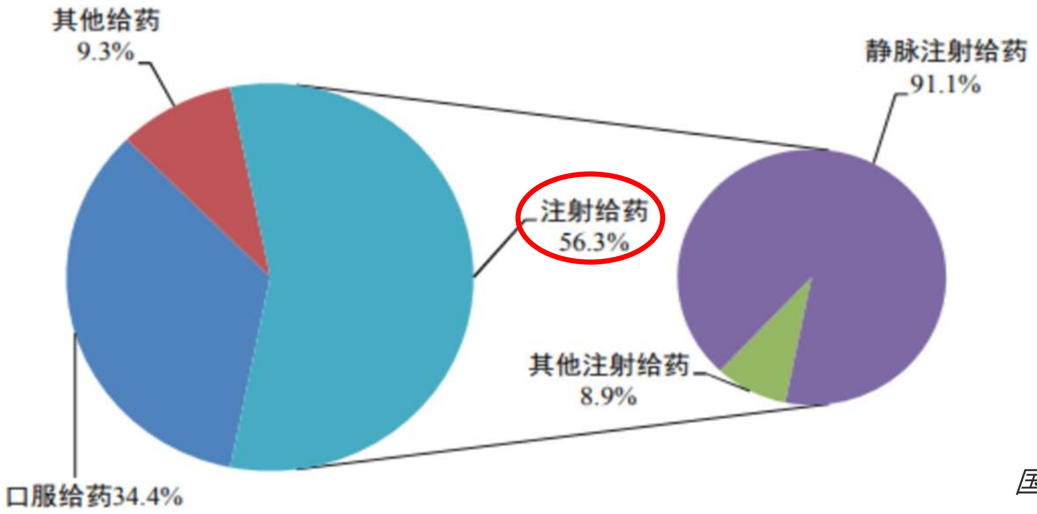

国家药品不良反应监测中心, 国家药品不良反应监测年度报告（2023年）.

政策支持

中华人民共和国中央人民政府
www.gov.cn

《关于持续做好抗菌药物临床应用管理有关工作的通知》国卫办医发〔2018〕9号

二、继续加强抗菌药物临床应用重点环节管理

（一）继续实施抗菌药物专档管理。在碳青霉烯类及替加环素等特殊使用级抗菌药物的基础上，鼓励有条件的地区在三级综合医院实行含酶抑制剂复合制剂的专档管理。将专档管理数据与药品采购记录、处方信息、临床病历信息等进行比对，理清所有纳入专档管理的抗菌药物购用情况，查找薄弱环节，采取有效措施，加强管理。

（二）进一步落实抗菌药物供应目录调整和备案管理要求。优化抗菌药物品种品规结构，及时将临床效果确切、经济性好、安全风险低的药品纳入供应目录，逐步淘汰药效药动力学特性差、不良反应多和循证医学证据不足的药品。正确认识β内酰胺类抗菌[illegible]

二（四）加强抗菌药物规范使用管理。“加强对二级医院和基层医疗机构的培训，提高其抗菌药物使用技术能力和管理水平，减少不合理的预防使用和静脉输注抗菌药物。”

（四）加强抗菌药物规范使用管理。鼓励制订本机构适用的感染性疾病临床路径和诊疗规范、指南。在继续关注Ⅰ类切口围手术期预防用药的基础上，加强Ⅱ类切口围手术期预防使用抗菌药物管理。加强对二级医院和基层医疗机构的培训，提高其抗菌药物使用技术能力和管理水平，减少不合理的预防使用和静脉输注抗菌药物。

政策支持

中华人民共和国中央人民政府
www.gov.cn

2021 年国家医疗质量安全改进目标

目标一　提高急性 ST 段抬高型心肌梗死再灌注治疗率
目标二　提高急性脑梗死再灌注治疗率
目标三　提高肿瘤治疗前临床 TNM 分期评估率
目标四　提高住院患者抗菌药物治疗前病原学送检率
目标五　提高静脉血栓栓塞症规范预防率
目标六　提高病案首页主要诊断编码正确率
目标七　提高医疗质量安全不良事件报告率
目标八　降低住院患者静脉输液使用率
目标九　降低血管内导管相关血流感染发生率
目标十　降低阴道分娩并发症发生率

2022 年国家医疗质量安全改进目标

目标一　提高急性 ST 段抬高型心肌梗死再灌注治疗率
目标二　提高急性脑梗死再灌注治疗率
目标三　提高肿瘤治疗前临床 TNM 分期评估率
目标四　提高住院患者抗菌药物治疗前病原学送检率
目标五　提高静脉血栓栓塞症规范预防率
目标六　提高感染性休克集束化治疗完成率
目标七　提高医疗质量安全不良事件报告率
目标八　降低非计划重返手术室再手术率
目标九　降低住院患者静脉输液使用率
目标十　降低阴道分娩并发症发生率

2023 年国家医疗质量安全改进目标

目标一　提高急性 ST 段抬高型心肌梗死再灌注治疗率
目标二　提高急性脑梗死再灌注治疗率
目标三　提高肿瘤治疗前临床 TNM 分期评估率
目标四　降低住院患者围手术期死亡率
目标五　提高医疗质量安全不良事件报告率
目标六　提高住院患者静脉输液规范使用率
目标七　提高四级手术术前多学科讨论完成率
目标八　提高感染性休克集束化治疗完成率
目标九　提高静脉血栓栓塞症规范预防率
目标十　降低阴道分娩并发症发生率

2024 年国家医疗质量安全改进目标

目标一　提高急性脑梗死再灌注治疗率
目标二　提高肿瘤治疗前临床 TNM 分期评估率
目标三　提高静脉血栓栓塞症规范预防率
目标四　提高感染性休克集束化治疗完成率
目标五　提高住院患者静脉输液规范使用率
目标六　提高医疗质量安全不良事件报告率
目标七　提高四级手术术前多学科讨论完成率
目标八　提高关键诊疗行为相关记录完整率
目标九　降低非计划重返手术室再手术率
目标十　降低阴道分娩并发症发生率

静脉输液和抗菌药物静脉使用原则

- **静脉输液指征：**

✓ 不存在脱水、可正常进食和口服药物的原则上不应静脉输液。

✓ 对于存在脱水情况、不能口服或不能耐受口服给药（如吞咽困难）、 存在可能明显影响口服药物吸收的情况（如呕吐、严重腹泻、胃肠道病变或肠道吸收功能障碍等）的患者可酌情给予静脉输液

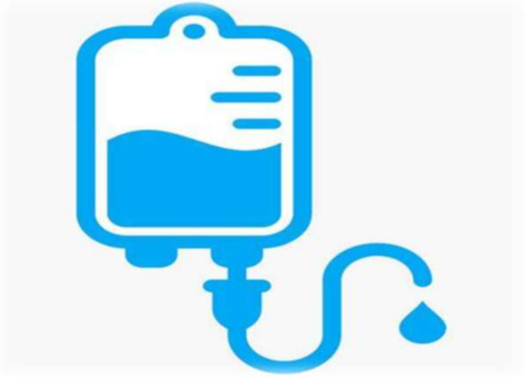

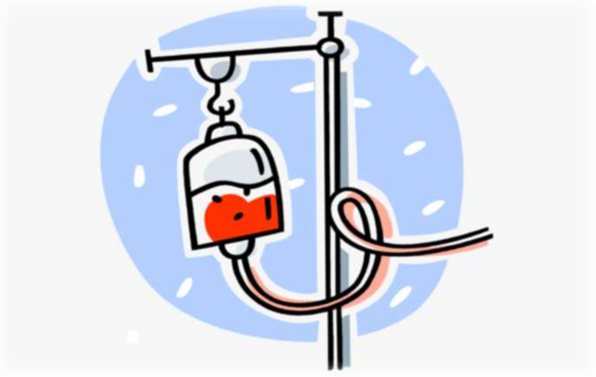

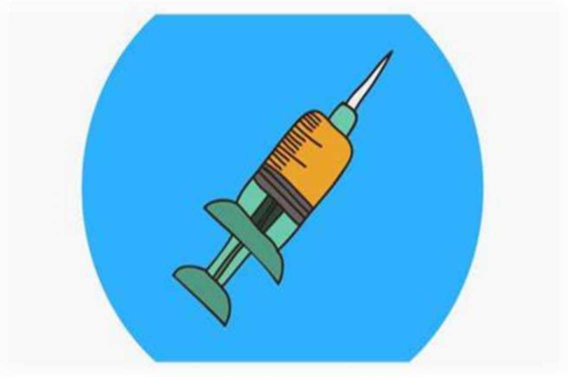

静脉输液和抗菌药物静脉使用原则

对于轻、中度感染的大多数患者，应予口服治疗，选取口服吸收良好的抗菌药物品种，不必采用静脉或肌内注射给药。仅在下列情况下可先予以注射给药：

①不能口服或不能耐受口服给药的患者（如吞咽困难者）

②患者存在明显可能影响口服药物吸收的情况（如呕吐、严重腹泻、胃肠道病变或肠道吸收功能障碍等）

③所选药物有合适抗菌谱，但无口服剂型

④需在感染组织或体液中迅速达到高药物浓度以达杀菌作用者（如感染性心内膜炎、化脓性脑膜炎等）

⑤感染严重、病情进展迅速，需给予紧急治疗的情况（如血流感染、重症肺炎等）

抗菌药物临床应用指导原则（2015年版）

静脉用药在体内的PK/PD

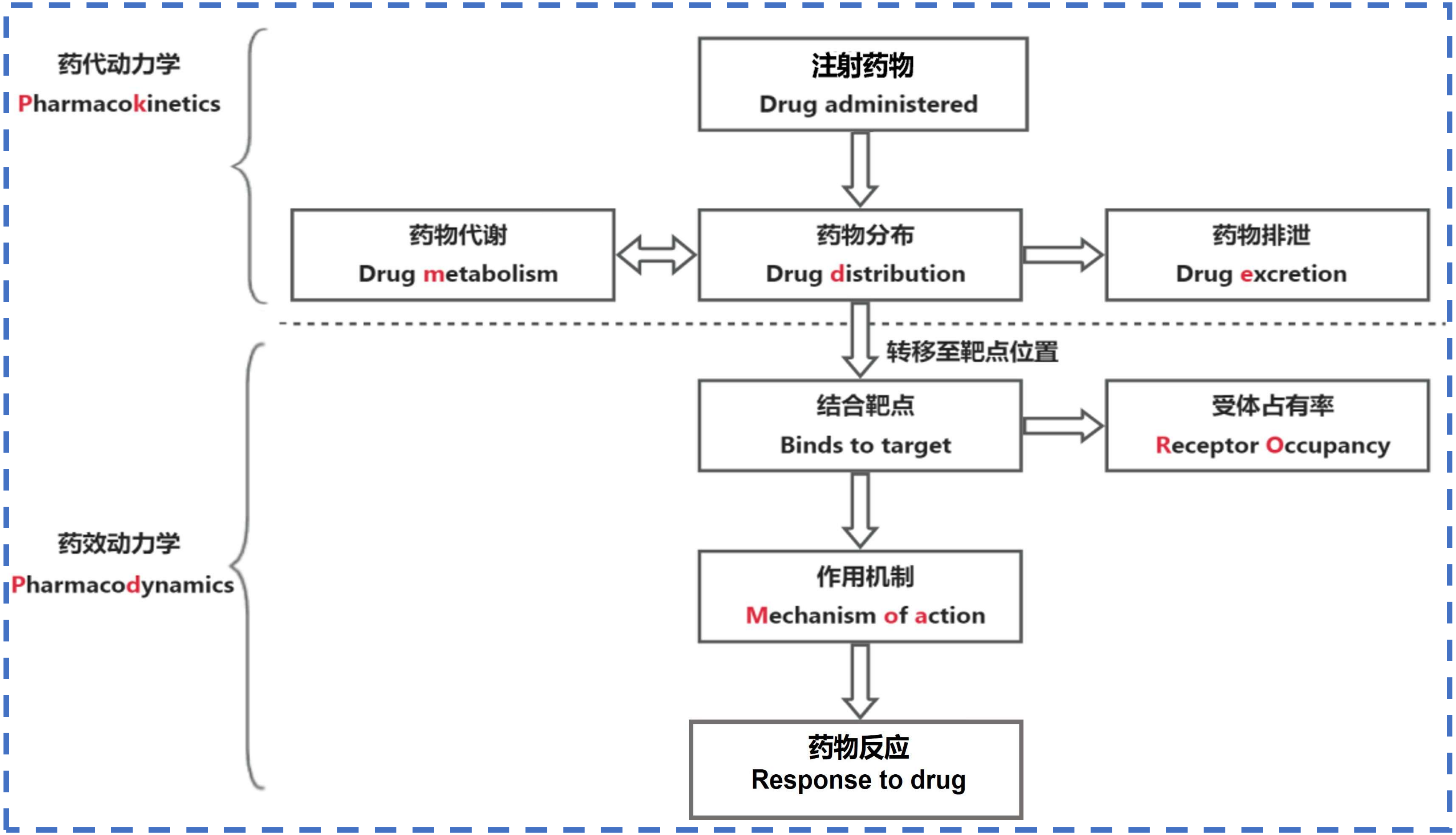

口服用药在体内的PK/PD

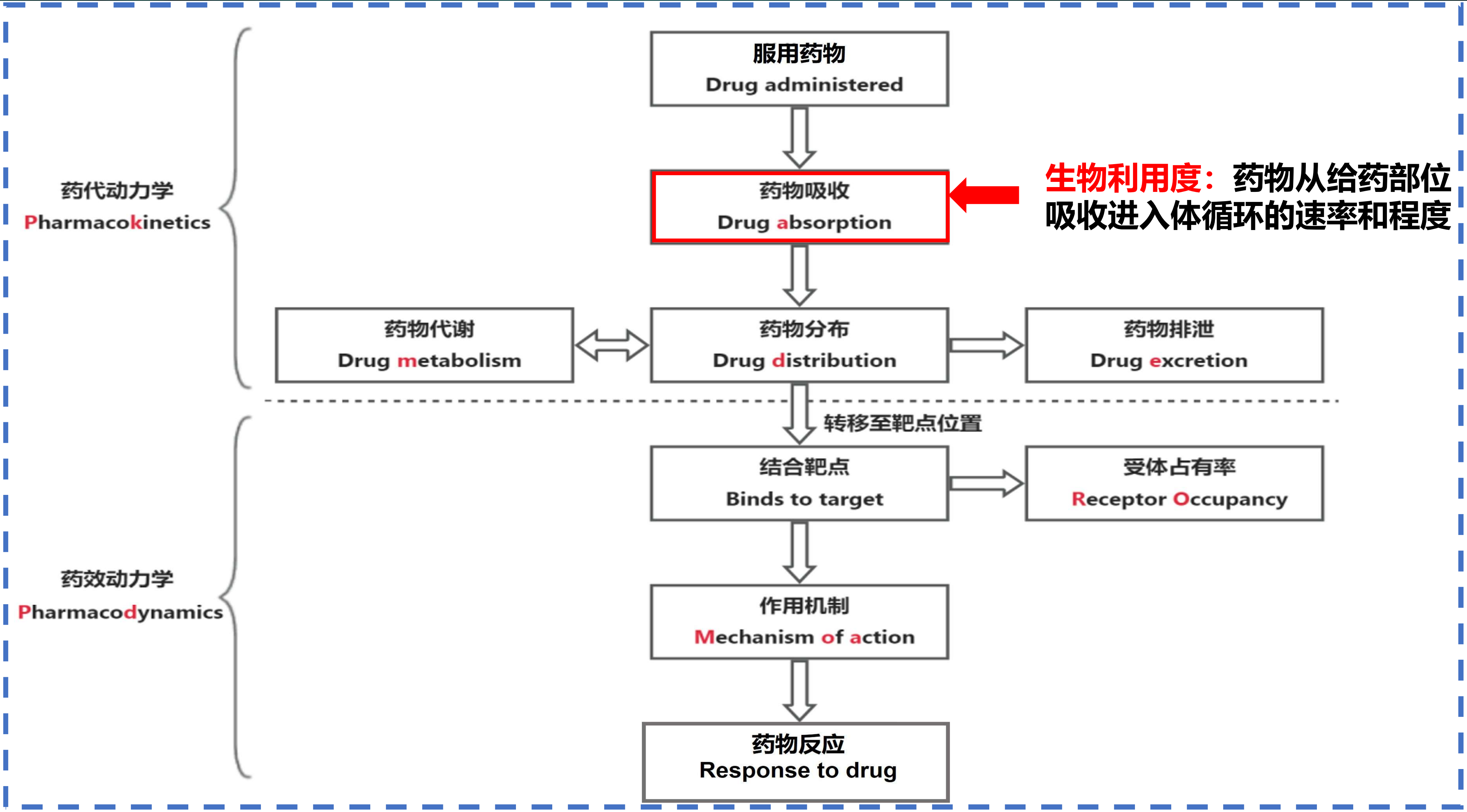

抗菌药物序贯疗法的临床药代动力学和药效学原则研究

2023年，Landersdorfer等人在临床药代动力学和药效学原则的背景下，研究早期从静脉注射改为口服抗生素的理由。

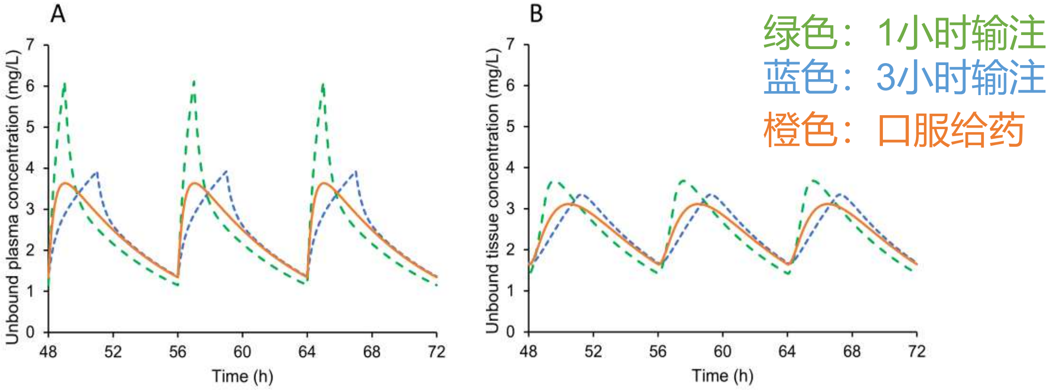

绿色：1小时输注
蓝色：3小时输注
橙色：口服给药

β-内酰胺类等时间依赖型抗生素效果评价：游离血浆浓度超过感染病原体最低抑菌浓度的时间百分比（%fT>MIC）

氟喹诺酮类和氨基糖苷类等浓度依赖型抗生素效果评价：游离血药浓度-时间曲线下面积与最低抑菌浓度的比值(fAUC/MIC)和最大游离抗生素血浆浓度与最低抑菌浓度的比值(fCmax/MIC)

模拟在零时间开始的静脉注射方案在48h后要么继续静脉输注（绿色和蓝色线），要么在48小时切换到口服方案（橙色线），血浆(A)和组织(间质液)(B)中抗生素的游离浓度-时间分布。

结 果： 在相同剂量和生物利用度的情况下，口服吸收导致血浆浓度增加较慢，比快速静脉注射获得的fCmax更低，口服后的fCmax通常更类似于3小时的输液。相比之下，组织中的fCmax预计在所有方案中都是相似的。

Landersdorfer CB, et al. Clinical pharmacological considerations in an early intravenous to oral antibiotic switch: are barriers real or simply perceived? Clin Microbiol Infect. 2023.IF: 14.2 Q1.

The NEW ENGLAND JOURNAL of MEDICINE

CURRENT ISSUE ∨ SPECIALTIES ∨ TOPICS ∨

ORIGINAL ARTICLE

Oral versus Intravenous Antibiotics for Bone and Joint Infection

Published January 30, 2019 | N Engl J Med 2019;380:425-436 | DOI: 10.1056/NEJMoa1710926

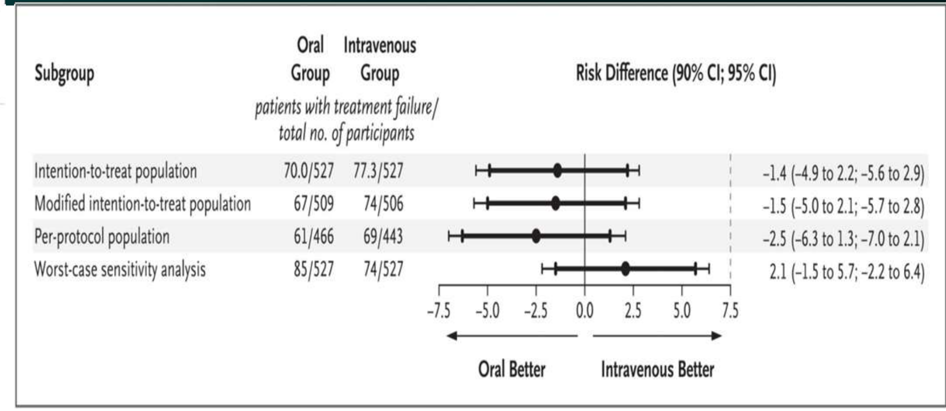

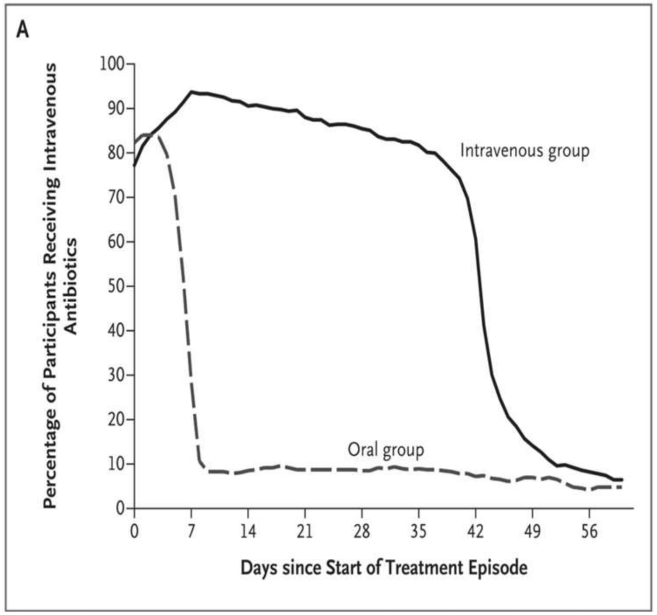

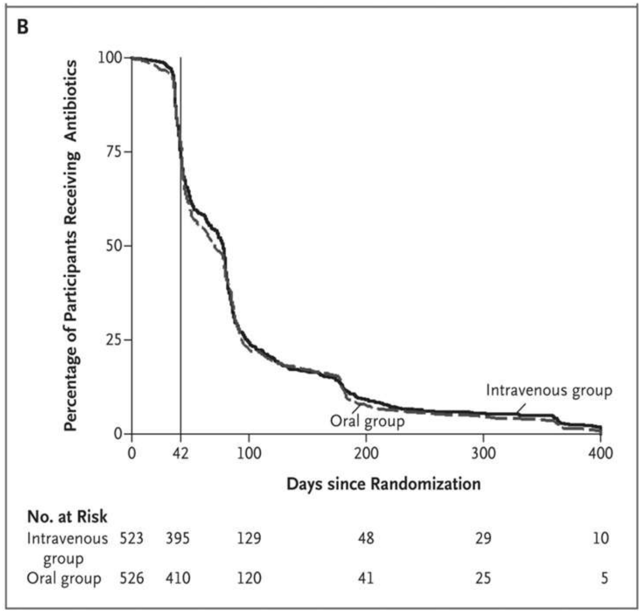

共入组1054例（每组527例），静脉组506例中有74例（14.6%）治疗失败，口服组509例中有67例（13.2%）失败，**严重不良事件发生率两组差异无统计学意义**（*P*=0.58）。**静脉组导管相关并发症显著增多**（9.4%vs1.0%）。

结论：对于复杂骨与关节感染，**在前6周使用口服抗生素治疗不劣于静脉抗生素治疗**，**1年随访的治疗失败率无显著差异。**

Randomized Controlled Trial > *JAMA* (IF: 45.54; Q1). 2021 Jan 26;325(4):353-362.

doi: 10.1001/jama.2020.23525.

Effect of Oral Moxifloxacin vs Intravenous Ertapenem Plus Oral Levofloxacin for Treatment of Uncomplicated Acute Appendicitis: The APPAC II Randomized Clinical Trial

FULL TEXT LINKS

JAMA FULL TEXT

FREE Full text PMC

ACTIONS

Cite

对于成人无并发症急性阑尾炎患者，**7天口服莫西沙星与2天静脉厄他培南序贯5天左氧氟沙星联合甲硝唑**的治疗方案均获得超过65%的成功率；在为期1年的随访期间，**口服抗生素单药治疗组的总体并发症发生率为4.8%，而静脉转口服抗生素组为7.3%。**

Figure 2. Time to Appendectomy After Initial Treatment in a Study of the Effect of Oral Moxifloxacin vs Intravenous Ertapenem Plus Oral Levofloxacin for Treatment of Uncomplicated Acute Appendicitis

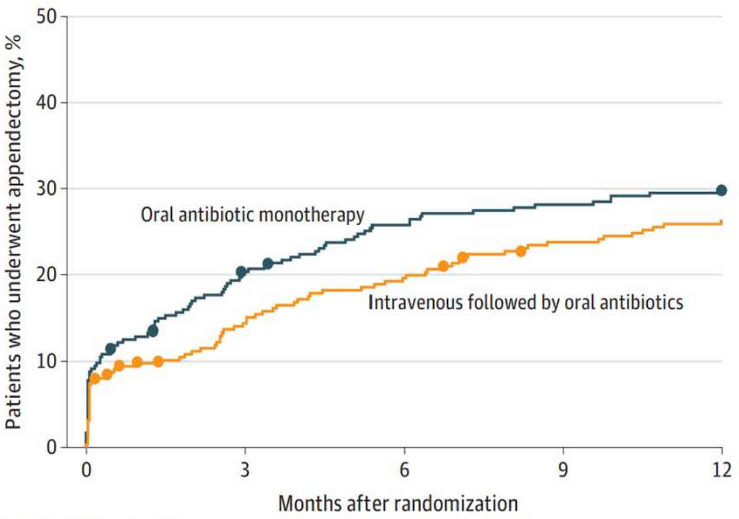

No. of patients at risk

	0	3	6	9	12
Oral antibiotic monotherapy	295	235	219	212	207
Intravenous followed by oral antibiotics	286	245	230	218	211

A total of 581 of 583 patients (99.7%) were followed up to achievement of the primary outcome or to 1 year and included in this post hoc analysis. The solid dots represent appendectomies of histologically normal appendixes.

Table 3. Adverse Events in a Study of the Effect of Oral Moxifloxacin vs Intravenous Ertapenem Plus Oral Levofloxacin for Treatment of Uncomplicated Acute Appendicitis

	No.	
Adverse event	Oral antibiotic monotherapy group (n = 295)	Intravenous followed by oral antibiotics group (n = 288)
Related to antibiotic treatment[a]	6	14
Skin eczema	3	3
Other allergic reaction	1	2
Tendinitis	1	1
Blurred vision	1	0
Prolonged diarrhea[b]	0	5
Candidiasis (oral or vaginal)	0	3
Tendon rupture	0	0
Related to operative treatment[a]	9	10
Abdominal pain, incisional pain, or obstructive symptoms	7	7
Surgical site infection	2	3
Incisional hernias	0	0
Other miscellaneous symptoms related to antibiotic treatment		
Nausea	23	40
Diarrhea	11	36
Metallic taste sensation	1	23
Patients with at least 1 adverse event, No./total No. (%) [95% CI][c]	14/295 (4.8) [2.3-7.2]	21/286 (7.3) [4.3-10.4]

常见抗菌药物口服生物利用度

类别	药名	生物利用	药名	生物利用
头孢类	阿莫西林	75%~90%	头孢呋辛酯（空腹）	37%
	阿莫西林+克拉维酸	60%	头孢呋辛酯（餐后）	52%
	头孢拉定	90%	头孢克洛（空腹）	93%
	头孢克肟	40%~50%	头孢克洛（餐后）	50%
硝基咪唑类	甲硝唑	80~90%	奥硝唑	> 90%
喹诺酮类	左氧氟沙星	> 90%	环丙沙星	70%~80%
	莫西沙星	> 90%	氧氟沙星	85%~95%
抗真菌类	氟康唑	90%	伏立康唑	96%

注：本表格整理自国家抗微生物治疗指南（第3版）；药品说明书，可能存在不同厂家药品剂量或口服吸收率不同，仅供参考。

“药学干预对急诊上呼吸道细菌感染患者抗菌药物输液率的影响”研究项目介绍

背景与现状

Multicenter Study > *JAMA Intern Med* (IF: 18.652; Q1). 2014 Dec;174(12):1914-20.
doi: 10.1001/jamainternmed.2014.5214.

FULL TEXT LINKS

FULL TEXT JAMA Internal Medicine

Use and prescription of antibiotics in primary health care settings in China

Figure. Antibiotic Use in the 4 Most Common Infectious Conditions of Outpatients

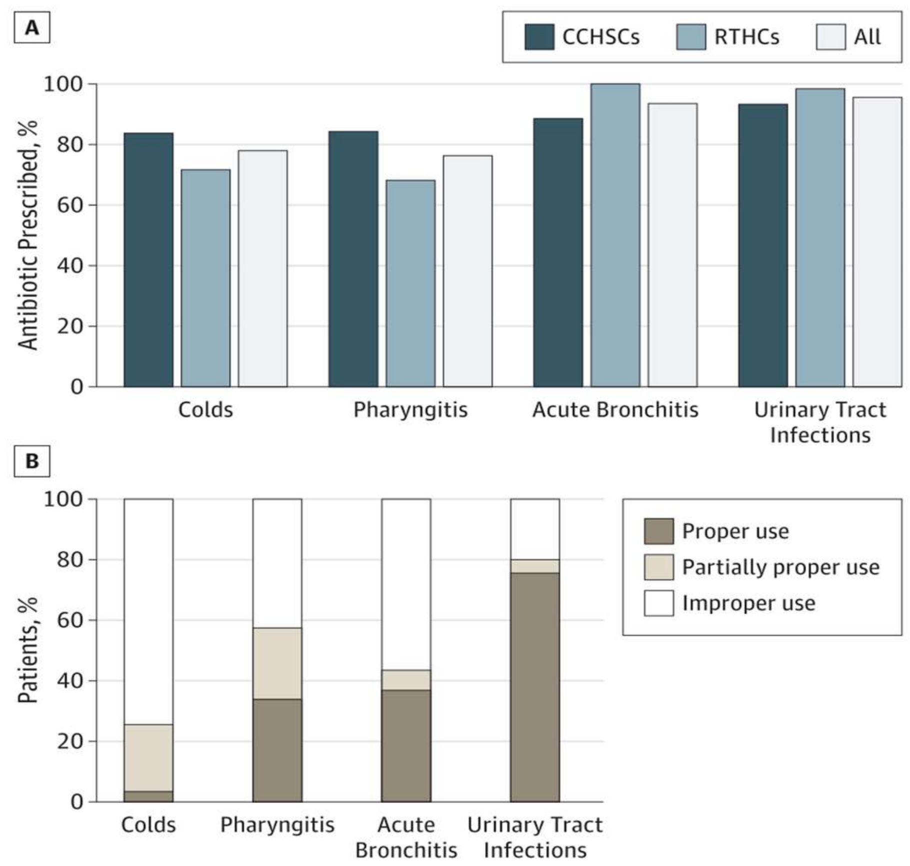

- 在7311份门急诊处方中，52.9%包含了抗菌药物；
- 对于**感冒**、**咽炎**、**急性支气管炎**和**尿路感染**，开具抗菌药物的比例很高，除了尿路感染外，其他处方大多数都不适宜。

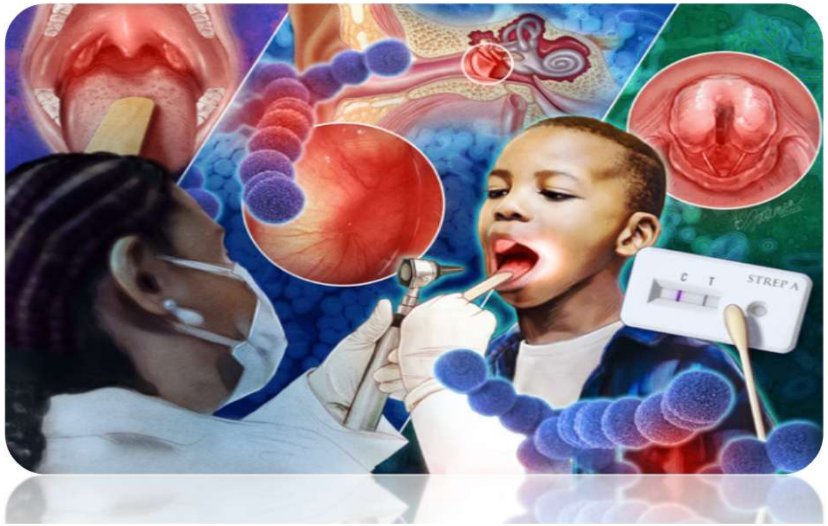

研究目的

本研究旨在遵循科学的给药原则前提下，提高医疗质量，保障患者安全，优化资源配置

- 明确药学干预是否能减少急诊上呼吸道细菌感染患者的**抗菌药物输液率**
- 分析药学干预后对上呼吸道细菌感染**患者转归**和**用药安全性**的影响

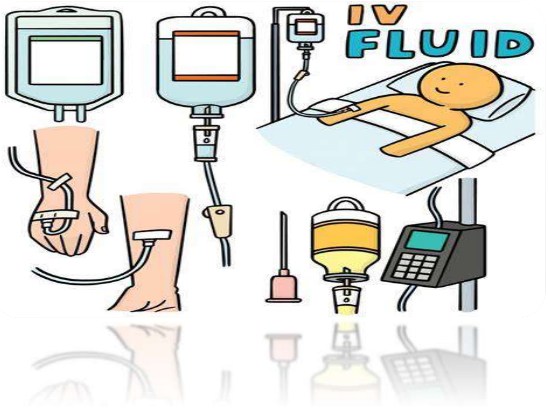

研究设计与方法

●**研究设计：**

多中心、整群随机对照研究

●**研究人群：**

18周岁及以上，性别不限，急诊就诊的上呼吸道细菌感染患者

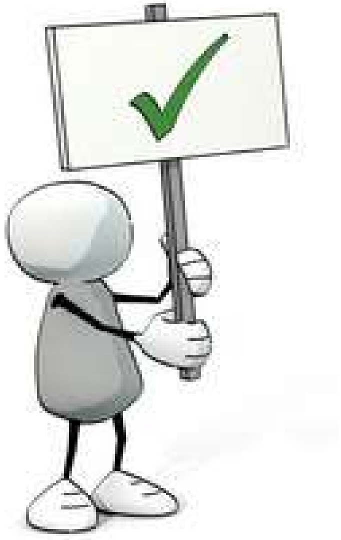

●患者入选标准（入选患者需同时满足以下四条标准）

- ①急诊就医的上呼吸道感染患者；
- ②有实验室检查，WBC $> 10\times10^{9}$/L或CRP≥10.0mg/L，需要使用抗菌药物者；
- ③≥18周岁；
- ④局部体征和症状至少满足以下一条：(1)发热，(2)咳嗽，(3)鼻炎（打喷嚏、鼻塞或流鼻涕），(4)咽炎（喉咙痛），(5)呼吸急促，(6)喘息，(7)胸痛。

●**患者排除标准**（**满足以下任一条件即需排除**）

➢①除上呼吸道感染外，同时合并其他感染的患者；

➢②根据医务工作者评估需住院治疗或需更高一级医疗机构治疗的患者；

➢③不能口服或胃肠道功能严重障碍的患者；

➢④特殊患者：粒缺患者、骨髓抑制患者、放化疗治疗期间患者、免疫抑制剂治疗患者、艾滋病人、先天性免疫功能缺陷病人、妊娠期患者、有精神疾病患者。

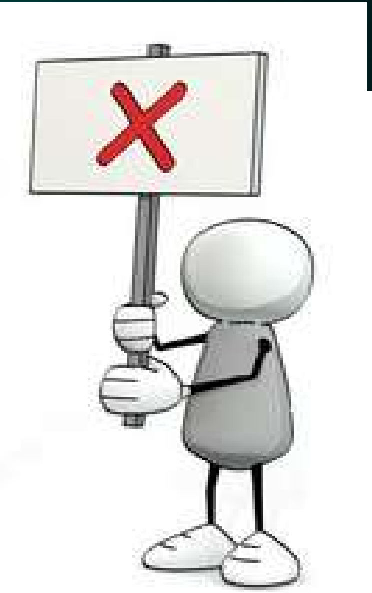

●干预与对照措施

➢**试验组：** 常规医疗服务+药师宣教服务

➢**对照组：** 常规医疗服务

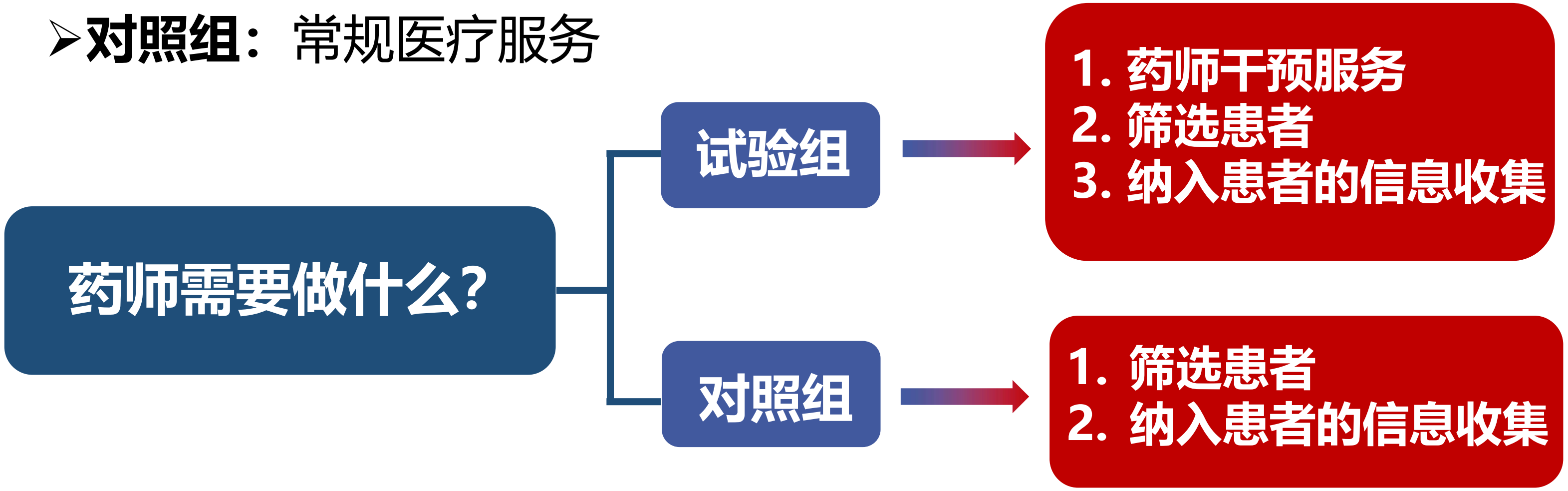

●随访计划

➢ 患者首次急诊**就诊结束后14天，各分中心需补充患者复诊信息**；

➢ 患者首次急诊**就诊结束后14天，**主中心使用标准化的随访问卷行**电话随访**

干预措施

- ➢**每月**初药师对医务人员（医生、护理）进行**抗菌药物宣教培训**；
- ➢给**医务人员**发放抗菌药物用药建议**口袋卡片**；
- ➢发放**患者用药教育宣传单**。

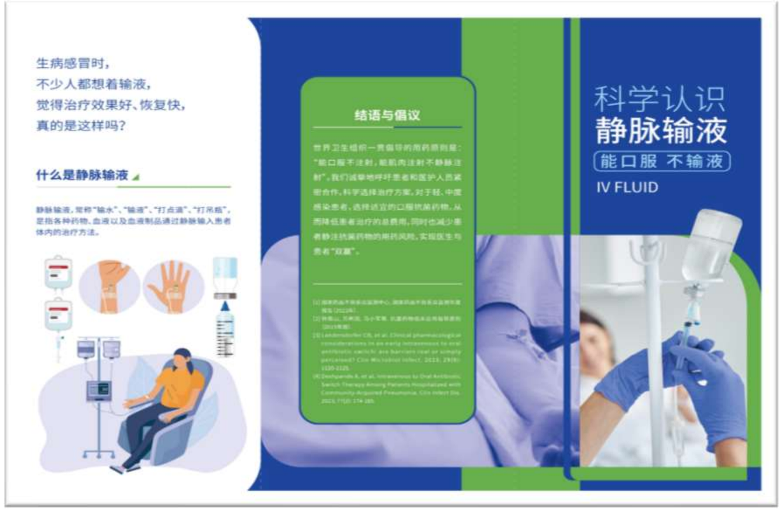

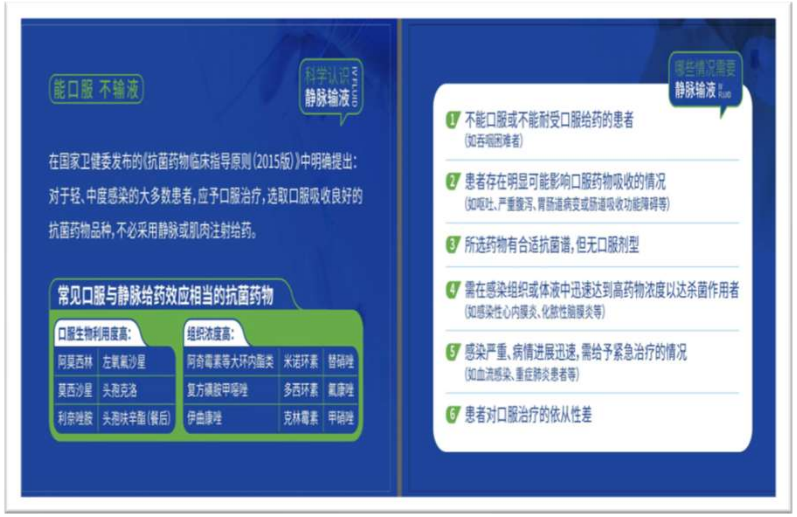

结局指标

	指标名称	指标定义
主要结局指标	抗菌药物输液率	抗菌药物针剂处方数/总抗菌药物处方数
次要结局指标	症状持续时间	患者上呼吸道感染症状的持续天数
	药物不良反应	合格药品在正常用法用量下出现的与用药目的无关的有害反应，本研究中特指抗菌药物
	病情加重或反复	14天内因任何原因再次接受静脉/口服抗菌药物治疗的比例
	病情加重或反复	患者14天内再次前往医疗机构门急诊就诊的比例
	病情加重	患者在14天内因本次急性呼吸道感染住院的比例

急诊常见上呼吸道感染

➢ 急性细菌性咽炎及扁桃体炎：

- ✓ 口服青霉素类（阿莫西林）、口服第一代或第二代头孢菌素（头孢呋辛）；
- ✓ 青霉素过敏患者可口服四环素类（多西环素）或氟喹诺酮类（左氧氟沙星、莫西沙星）

➢ 急性气管-支气管炎：

- ✓ 以病毒感染多见，多数病例为自限性，不应常规使用抗菌药物；
- ✓ 可能由肺炎支原体或百日咳博德特菌引起者，可采用大环内酯类、四环素类或氟喹诺酮类；
- ✓ 肺炎衣原体感染可用多西环素、大环内酯类或氟喹诺酮类

举例一

[redacted] / 29岁 / 女 / 42.0kg

收起

诊断：咳嗽 | 呼吸道感染

过敏：

患者号：[redacted]　　处方号：7114011423

西/成药　检验　手术　　HALO...　显示设置

组号	药品名称	给药剂量	给药频率	给药途径
1	盐酸氨溴索注射液<br>盐酸氨溴索注射液(科伦)	30mg	once<br>ONCE	副药<br>副药
	氯化钠注射液<br>0.9%氯化钠注射液(非PVC)	100ml	once<br>ONCE	静脉滴注<br>静脉滴注
2	注射用阿奇霉素<br>注射用阿奇霉素(巴珠)	0.5g	once<br>ONCE	副药<br>副药
	葡萄糖注射液<br>(5%)葡萄糖注射液	250ml	once<br>ONCE	静脉滴注<br>静脉滴注

举例二

[redacted] / 33岁 / 男 / 70.0kg

收起

诊断：咽痛

过敏：注射用头孢曲松钠(罗氏芬)1g*1瓶|阿莫西林胶囊(诺莫灵)

患者号：[redacted]　　处方号：2114393167

西/成药　检验　手术　　HALO...　显示设置

组号	药品名称	给药剂量	给药频率	给药途径
0	盐酸莫西沙星片<br>盐酸莫西沙星片(言宁)	1片	1次/天<br>QD	口服<br>口服(餐后)
	左氧氟沙星氯化钠注射液<br>左氧氟沙星氯化钠注射液(新昌)	0.5g	1次/天<br>QD	静脉滴注<br>静脉滴注

举例三

[redacted] / 38岁 / 男 / 80.0kg

收起

诊断：呼吸道感染

过敏：

患者号：[redacted]　　处方号：0113722291

西/成药　检验　手术　　HALO...　显示设置

组号	药品名称	给药剂量	给药频率	给药途径
0	左氧氟沙星氯化钠注射液<br>左氧氟沙星氯化钠注射液(新昌)	0.5g	1次/天<br>QD	静脉滴注<br>静脉滴注

不必要的静脉使用抗菌药物

- **口服生物利用度高、组织或血药浓度能达到理想治疗浓度的抗菌药物**

口服生物利用度极高：左氧氟沙星、莫西沙星、利奈唑胺

组织浓度高：阿奇霉素等大环内酯类、克林霉素、氯霉素、多西环素、米诺环素、SMZ-TMP、硝基咪唑类、氟康唑、伊曲康唑等

- 以上抗菌药物即使中重度感染，口服用药效果与静脉使用也基本一致

感谢各位老师的支持，

大家携手促进合理用药

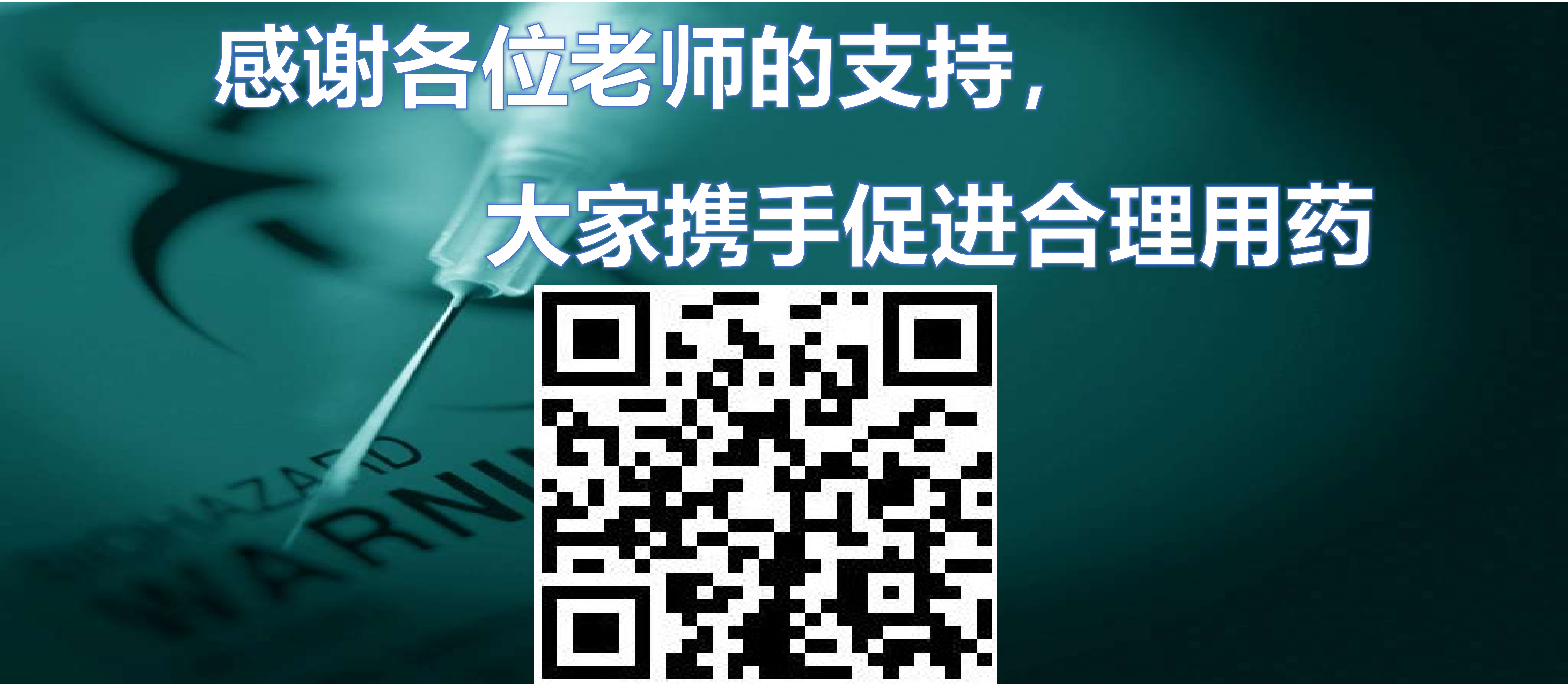

能口服不肌注，能肌注不输液！
