# Supplementary material for: Impact of pharmaceutical intervention on the use of intravenous antibiotics in patients with bacterial upper respiratory tract infections: protocol for a cluster-randomized controlled trial
Source: Front Public Health. 2026 Mar 10;14:1742217. doi: 10.3389/fpubh.2026.1742217 (PMC13008920; doi:10.3389/fpubh.2026.1742217)
Supplement: Supplementary file 2 [file Data_Sheet_2.PDF]

能口服 不输液

科学认识
静脉输液
IV FLUID

在国家卫健委发布的《抗菌药物临床指导原则（2015版）》中明确提出：对于轻、中度感染的大多数患者，应予口服治疗，选取口服吸收良好的抗菌药物品种，不必采用静脉或肌肉注射给药。

常见口服与静脉给药效应相当的抗菌药物

口服生物利用度高：

阿莫西林	左氧氟沙星
莫西沙星	头孢克洛
利奈唑胺	头孢呋辛酯（餐后）

组织浓度高：

阿奇霉素等大环内酯类	米诺环素	替硝唑
复方磺胺甲噁唑	多西环素	氟康唑
伊曲康唑	克林霉素	甲硝唑

哪些情况需要静脉输液 IV FLUID

1. 不能口服或不能耐受口服给药的患者
（如吞咽困难者）

2. 患者存在明显可能影响口服药物吸收的情况
（如呕吐、严重腹泻、胃肠道病变或肠道吸收功能障碍等）

3. 所选药物有合适抗菌谱，但无口服剂型

4. 需在感染组织或体液中迅速达到高药物浓度以达杀菌作用者
（如感染性心内膜炎、化脓性脑膜炎等）

5. 感染严重、病情进展迅速，需给予紧急治疗的情况
（如血流感染、重症肺炎患者等）

6. 患者对口服治疗的依从性差
