# Supplementary material for: Impact of pharmaceutical intervention on the use of intravenous antibiotics in patients with bacterial upper respiratory tract infections: protocol for a cluster-randomized controlled trial
Source: Front Public Health. 2026 Mar 10;14:1742217. doi: 10.3389/fpubh.2026.1742217 (PMC13008920; doi:10.3389/fpubh.2026.1742217)
Supplement: Supplementary file 3 [file Data_Sheet_3.PDF]

生病感冒时，
不少人都想着输液，
觉得治疗效果好、恢复快，
真的是这样吗？

什么是静脉输液

静脉输液，常称“输水”、“输液”、“打点滴”、“打吊瓶”，是指各种药物、血液以及血液制品通过静脉输入患者体内的治疗方法。

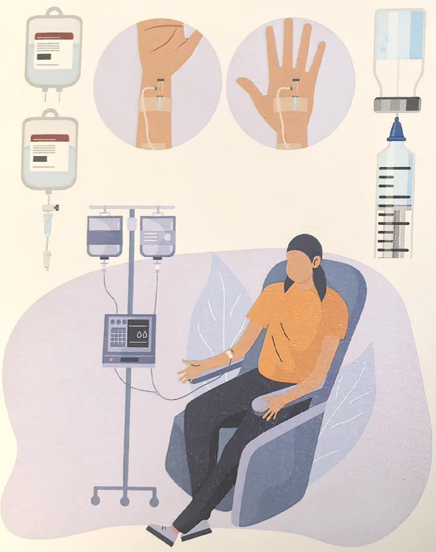

结语与倡议

世界卫生组织一贯倡导的用药原则是：“能口服不注射，能肌肉注射不静脉注射”。我们诚挚地呼吁患者和医护人员紧密合作，科学选择治疗方案，对于轻、中度感染患者，选择适宜的口服抗菌药物。降低患者治疗费用的同时也减少患者静注抗菌药物的用药风险，实现医生与患者“双赢”。

[1] 国家药品不良反应监测中心, 国家药品不良反应监测年度报告(2023年).
[2] 钟南山, 万希润, 马小军等. 抗菌药物临床应用指导原则(2015年版).
[3] Landersdorfer CB, et al. Clinical pharmacological considerations in an early intravenous to oral antibiotic switch: are barriers real or simply perceived? Clin Microbiol Infect. 2023; 29(9): 1120-1125.
[4] Deshpande A, et al. Intravenous to Oral Antibiotic Switch Therapy Among Patients Hospitalized with Community-Acquired Pneumonia. Clin Infect Dis. 2023; 77(2): 174-185.

科学认识
静脉输液

能口服 不输液

IV FLUID

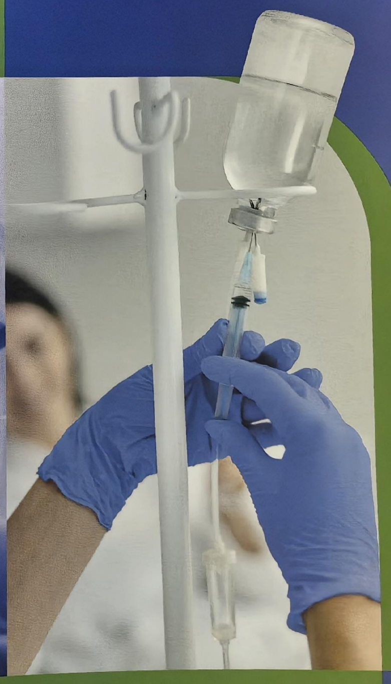

哪些情况需要静脉输液

当您出现吞咽困难时，通过静脉输液将药物直接送入血液循环，可以避免您因吞咽困难而无法服药的问题；

当您存在严重的吸收障碍时，通过静脉输液给药可以绕过肠道，确保药物被有效吸收；

当出现经口服或肌注给药治疗无效的疾病，或者出现病情危重、发展迅速的情况时，静脉输液能迅速将药物送达全身，提高药物在组织中的浓度，才能更好地控制病情。

抗菌药物：选择口服还是输液？

抗菌药物是用来治疗细菌真菌感染的一类药物，它能够抑制或杀灭细菌、真菌，用于预防和治疗细菌、真菌感染。我们常见的如：青霉素、头孢菌素类、阿奇霉素、四环素、左氧氟沙星等都属于抗菌药物。

在国家卫健委发布的《抗菌药物临床应用指导原则（2015版）》中明确提出：**对于轻、中度感染的大多数患者，应予口服治疗，选取口服吸收良好的抗菌药物品种，不必采用静脉或肌肉注射给药。**

口服和输液的区别其实就是吸收入血的速度不一样。口服是较慢也是较温和的一种形式，而静脉输液是相对最快的，只要体内药物浓度达标，两种方法就是殊途同归。

常见口服与静脉给药效应相当的抗菌药物推荐如下：

口服生物利用度高：
左氧氟沙星、莫西沙星、利奈唑胺、阿莫西林、头孢克洛、头孢呋辛酯（餐后）

组织浓度高：
阿奇霉素等大环内酯类、克林霉素、多西环素、米诺环素、复方磺胺甲噁唑、甲硝唑、替硝唑、氟康唑、伊曲康唑等。

滥用静脉输液的危害

1.不良反应发生率增加

根据2023年《国家药品不良反应监测年度报告》显示，2023年药品不良反应/事件报告中，注射给药占56.3%，其中静脉注射给药占注射给药的91.1%

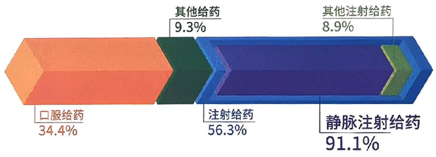

2.静脉炎

药物的浓度、渗透压、pH值、滴速、给药持续时间、留置针的长时间应用，都可能刺激您的血管，导致静脉炎。

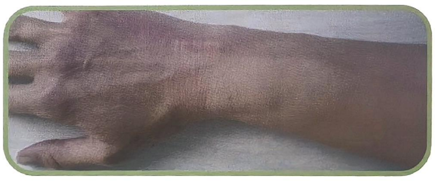

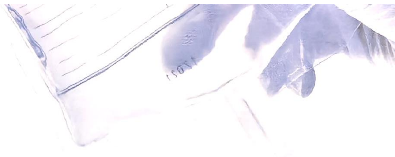

3.微粒栓塞

静脉输注中药物配伍不合理、安瓿折断和针筒穿刺橡胶塞等，都可能产生或混入微粒，引起重要器官的小血管堵塞，微循环障碍及肉芽肿的形成。

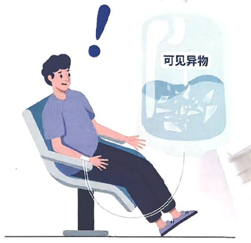

4.空气栓塞

输液器中若有空气未排尽，一旦过多空气进入静脉，可能导致您产生胸闷、呼吸困难等严重症状。

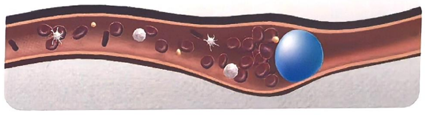

5.更易产生耐药性

长期反复应用抗菌药物尤其是注射剂，当剂量不足时，特别容易诱导病原体产生耐药性。

6.其他

过度使用静脉输液，会增加您的心理负担和时间成本，带来额外的经济压力；同时还可能提高医院的医疗成本、浪费医疗资源。
